# Assessing the suitability of lakes and reservoirs for recreation using Landsat 8

**DOI:** 10.1007/s10661-023-11830-5

**Published:** 2023-10-21

**Authors:** Darryl J. Keith, Wilson Salls, Blake A. Schaeffer, P. Jeremy Werdell

**Affiliations:** 1 Center of Environmental Measurement & Modeling, Office of Research and Development, US Environmental Protection Agency, Narragansett, RI 02882 USA; 2 Center of Environmental Measurement & Modeling, Office of Research and Development, US Environmental Protection Agency, Research Triangle Park, Durham, NC 27711 USA; 3 Ocean Ecology Laboratory, NASA Goddard Space Flight Center, Greenbelt, MD 20771 USA

**Keywords:** Secchi depth, Water clarity, Landsat 8, Quasi-analytical algorithm, Lakes

## Abstract

Water clarity has long been used as a visual indicator of the condition of water quality. The clarity of waters is generally valued for esthetic and recreational purposes. Water clarity is often assessed using a Secchi disk attached to a measured line and lowered to a depth where it can be no longer seen. We have applied an approach which uses atmospherically corrected Landsat 8 data to estimate the water clarity in freshwater bodies by using the quasi-analytical algorithm (QAA) and Contrast Theory to predict Secchi depths for more than 270 lakes and reservoirs across the continental US. We found that incorporating Landsat 8 spectral data into methodologies created to retrieve the inherent optical properties (IOP) of coastal waters was effective at predicting in situ measures of the clarity of inland water bodies. The predicted Secchi depths were used to evaluate the recreational suitability for swimming and recreation using an assessment framework developed from public perception of water clarity. Results showed approximately 54% of the water bodies in our dataset were classified as “marginally suitable to suitable” with approximately 31% classed as “eminently suitable” and approximately 15% classed as “totally unsuitable–unsuitable”. The implications are that satellites engineered for terrestrial applications can be successfully used with traditional ocean color algorithms and methods to measure the water quality of freshwater environments. Furthermore, operational land-based satellite sensors have the temporal repeat cycles, spectral resolution, wavebands, and signal-to-noise ratios to be repurposed to monitor water quality for public use and trophic status of complex inland waters.

## Introduction

The USEPA National Lakes Assessment Report estimated that there are more than 300,000 lakes, ponds, and reservoirs in the conterminous US that provide important recreational, esthetic, and public health benefits (USEPA, 2017). Water quality is a critical consideration in determining the beneficial uses of these waters. Water clarity has long been used by aquatic monitoring programs as a visual indicator of the condition of water quality. The clarity of water is a measure of how far down light can penetrate through the water column (Kirk, 1996). This characteristic has important implications for the diversity and productivity of aquatic life that can be supported in freshwater and marine systems. Clear waters, for example, are characterized by low concentrations of suspended soil particles and algae which allow more sunlight to reach benthic flora and fauna such as submerged aquatic vegetation (SAV). However, studies (e.g., Tzortziou et al., 2015) have shown that the attenuation of sunlight by colored dissolved organic matter (CDOM) plays a distinctive role in reducing the clarity of water bodies, even those with low algal concentrations and suspended sediment concentrations.

Clear waters are generally valued for esthetic and recreational purposes. Several studies have established the public’s likelihood to recreate at water bodies based on their perception of water quality as determined by the clarity of local waters (Angradi et al., 2018; Smith et al., 1995; Smith & Davies-Colley, 1992). A decline in lake-water clarity translates to a decline in property values (Gibbs et al., 2002), and improvements in clarity result in property price increases (Michael et al., 1996). Turbid waters, on the other hand, are marked by high levels of inorganic suspended sediments, which may smother near-shore habitats, bury benthic communities, and produce excessive amounts of anthropogenic nutrients resulting in algal blooms that cloud visibility by absorbing and scattering light. Water clarity is affected by several physical, chemical, and biological factors that are connected to the natural geology and human use of the surrounding watershed (Betz & Howard, 2009). Primary controlling factors are algae, non-algal suspended matter, and colored dissolved organic matter (CDOM) which alters the quality and quantity of light available for photosynthesis (Thane et al., 2014).

Lakes and reservoirs are optically complex environments that are routinely monitored by federal and state agencies, tribal nations, and citizen-volunteer groups for changes in water quality and water clarity (Rich et al., 2019; Topp et al., 2020; USEPA, 2017). These data are used by environmental managers to support resource-management decisions. During field operations, water clarity is often monitored using an inexpensive Secchi disk (Tyler, 1968). The Secchi disk is a 20-cm (8 in) diameter metal or weighted plastic disk, normally black and white, which is attached to a measured line and lowered into a lake until it can be no longer seen. The depth at which it disappears, known as the Secchi depth (*Z*_SD_; m^−1^), is inversely proportional to the light attenuation along the line of sight in the water column, which is controlled by the amount of organic and inorganic substances along this line of sight (Preisendorfer, 1986). Because of this relationship, *Z*_SD_ becomes a visual measure of water clarity.

While the equipment and process to collect this information are relatively inexpensive, extending this type of monitoring to the thousands of lakes in the conterminous US is logistically prohibitive. Water-quality monitoring efforts are often overseen by a single management agency that is usually constrained by personnel availability and limited financial resources. New tools are needed to facilitate the development of reliable and cost-effective monitoring programs at lake, watershed, state, and regional and national scales to assist in timely and consistent water clarity assessments.

Satellite visible-to-near infrared spectroradiometers provide a capability to monitor water clarity for resolvable lakes and reservoirs (Lee et al., 2016). The ability by satellites to resolve water bodies is based on the water-body size and shape as well as the spatial resolution of independent satellite sensors (Papenfus et al., 2020). Note that ocean-color multispectral sensors generally have pixel resolutions, from 300 m to 1 km, that limit their suitability for monitoring smaller inland water bodies.

Historically, the primary research goals of inland water remote sensing have been data collection, image processing, and algorithm development and calibration (Topp et al., 2020). During the last 10–15 years, remote sensing has emerged as a powerful analytical tool to monitor inland water bodies as satellite sensors have been equipped with more spectral bands and are refined to better spatially resolve lakes and reservoirs while in orbit. Data products and results have been used to identify spatiotemporal trends, drivers, and the impacts of changing inland water quality on ecosystem functions and human populations. Topp et al. (2020) concluded that this expansion has led to a better understanding of inland water processes because research results have better informed the broader inland-water scientific literature.

The Operational Land Imager (OLI) sensor onboard the NASA US Geological Survey (USGS) Landsat 8 (L8) satellite was designed to overcome some of the challenges of retrieving data from inland waters (Olmanson et al., 2016; Pahlevan et al., 2014; Pahlevan et al., 2017). OLI provides multispectral data at a 30-m pixel resolution, which may provide data for monitoring 275,897 (100%) US lakes and reservoirs at single-pixel resolution or 170,240 (62%) lakes and reservoirs using a 3 × 3-pixel grid (Clark et al., 2017). The potential strengths and limitations of the Landsat 7 and 8 for water clarity and colored dissolved organic matter (CDOM) measurement were evaluated in MN lakes using spectral data retrieved for summer of 2013 and 2014 and regionally tuned models (Olmanson et al., 2016). Results showed that the best water-clarity model for Landsat 8 used OLI band 2, band 4, and band 1. Olmanson et al. (2016) concluded that the OLI sensor’s improved ultra-blue and narrower near infrared (NIR) bands, along with improved radiometric and signal to noise ratios, seemed to provide substantial improvements that could create opportunities for accurately measuring and mapping CDOM and understanding the controls over water clarity and carbon cycles at regional to global scales.

We converted satellite-derived water clarity to Secchi depth to categorize the suitability of lakes for swimming and other public recreation based on survey criteria found in Smith et al. (1995) and Smith and Davies-Colley (1992). In this study we used the improved spectral capabilities of the OLI sensor to address the following questions:

1. Can *Z*_SD_ be accurately determined for inland lakes and reservoirs at the coterminous scales from OLI derived inherent optical properties and light attenuation character of freshwater lakes and reservoirs?
2. Can satellite *Z*_SD_ be used in an assessment framework to supplement social surveys which seek to assess the suitability of freshwater lakes and reservoirs for public recreation?

## Materials and methods

### Data acquisition

For this study, in situ *Z*_SD_ data from eutrophic, mesotrophic, and oligotrophic lakes and reservoirs were retrieved from the AquaSat database. AquaSat is the largest database of its kind with more than 600,000 field observations of total suspended sediments, dissolved organic carbon, chlorophyll *a* and Secchi depths from the Water Quality Portal (WQP) and LAGOS-NE databases, which are paired (matched) with Landsat archived images collected within 1–3 days of a sampling event (Ross et al., 2019). Match-up observations were developed using open source, R, and Python software (Ross et al., 2019).

The Aquasat database was queried to identify Landsat 8 images with less than 10% cloud cover and in situ *Z*_SD_ sample dates that occurred within 1–3 days of an overpass. Query results ranged from March 2013 to August 2018 with data files and associated metadata available for download in CSV format.

Sample dates retrieved from the Aquasat database query were used to search the USGS EarthExplorer website (earthexplorer.usgs.gov) for Landsat 8 level-2 collection (surface reflectance) images. Landsat 8 surface reflectance (SR) products provide an estimate of the surface spectral reflectance as it would be measured at ground level with Rayleigh atmospheric scattering or absorption removed (Ita.cr.usgs.gov/L8Level2SR). SR products are generated at the USGS Earth Resources Observation and Science (EROS) Center at a 30-m spatial resolution.

There were a variety of trade-offs considered with the use of EROS SR products including potential impacts from glint and greater remotely sensed reflectance (*R*_rs_) values when compared against traditional aquatic atmospheric correction approaches, but with potentially less impact due to land adjacency effects (Kuhn et al., 2019). An added advantage of the SR products is that the standardized approach is more readily accessible to water resource managers. The limited field study by Kuhn et al. (2019) indicated that the SR products were in best agreement with field measures along the Amazon River.

After the Rayleigh-corrected SR product was downloaded, the ACOLITE program was used to remove aerosol scattering effects and calculate *R*_rs_. ACOLITE bundles the atmospheric correction algorithms and processing software developed at RBINS (Royal Belgian Institute of Natural Sciences) for aquatic applications of satellite data, including Landsat (5/7/8) and Sentinel-2 (A/B) (Vanhellemont & Ruddick, 2015, 2016).

### Estimating the inherent optical properties of turbid lakes and reservoirs

Inherent optical properties (IOPs) are the absorption (*a*; m^−1^) and backscatter (*b*_b_; m^−1^) characteristics of a waterbody which are based on in situ properties determined independently of the position of the sun (Preisendorfer, 1961). In contrast, *R*_rs_ is an apparent optical property whose value is dependent on the angle of the sun. In this study, *R*_rs_ values derived from Landsat 8 OLI spectral data were used in the quasi-analytical algorithm (QAA) to derive IOP estimates. The QAA was developed for use in coastal and estuarine environments and is one of the most widely used algorithms for deriving inherent optical properties from *R*_rs_ (Lee et al., 2002, 2005). The equations used to transform *R*_rs_ into IOP values are found in QAA version 5 (QAA_v5; Lee et al., 2015) from the International Ocean Color Coordinating Group (IOCCG).

The QAA process starts with an estimation of total absorption (*a*_t_) at a reference wavelength (*λ*_0_) to be chosen from either 550, 555, or 560 nm. Based on *a*_t_ for *λ*_0_, total absorption is calculated at 443, 490, and 667 nm. These wavelengths corresponded to those of the SeaWiFS, MODIS, and MERIS ocean color sensors (Lee et al., 2015). For satellite sensors that do not have a 667 band, QAA_v5 does include a provision to derive *R*_rs_ (667). The OLI sensor on Landsat 8 has spectral bands generally comparable to the previously mentioned ocean color sensors; however, there is no 667 nm band.

### Estimating diffuse attenuation (K_d_) and Z_SD_ from the inherent properties of turbid lakes and reservoirs

The *R*_rs_ values calculated by ACOLITE were transformed into subsurface reflectances (*r*_rs_) using Lee et al. (2002). The *r*_rs_ values were used to estimate total absorption (*a*) at a wavelength (λ) and total backscatter (b_b_ (λ)) using Lee et al. (2016). According to Lee et al. (2013), the diffuse attenuation coefficient (*K*_d_) can be modeled as a function of *a*(*λ*) and *b*_b_ (*λ*). Based on these relationships, Secchi depths were derived from Lee et al. (2015):

1 $${Z}_{\textrm{SD}}=\frac{1}{2.5\operatorname{Min}\left({K}_{\textrm{d}}\right)}\ln \left[\left(\frac{t^2}{n^2}\right)\frac{\left|{r}_{\textrm{T}}-{r}_{\textrm{w}}^{\textrm{pc}}\right|}{c_{\textrm{t}}\left(0-\right)}\right]$$

- where Min(*K*_d_) = minimum diffuse attenuation at the transparent window within the visible domain (400–700 nm; Lee et al., 2015, 2016)
- $$\left(\frac{t^2}{n^2}\right)=\frac{\textrm{radiance}\ \textrm{transmittance}\ \textrm{across}\ \textrm{the}\ \textrm{water}-\textrm{air}\ \textrm{interface}}{\textrm{refractive}\ \textrm{index}\ \textrm{of}\ \textrm{water}}$$ = 0.52 (Mobley, 1994)
- *r*_T_ = downwelling irradiance just below the water surface = 0.27 (Preisendorfer, 1986)
- *r*_w_^pc^ = subsurface reflectance ≡ *r*_rs_
- *c*_t_ (0−) = contrast threshold for sighting a Secchi disk below the water surface = Weber contrast (Johnsen et al., 2011)

The contrast threshold was approximated using the attenuation coefficient of the transparent window (minimum *k*_d_; Lee et al., 2015) and the absorption and backscatter characteristics of the water column:

2 $${c}_t\left(0-\right)=\frac{\operatorname{Min}\left({k}_{\textrm{d}}\right)\left(a+{b}_{\textrm{b}}\right)}{\left(a+{b}_{\textrm{b}}\right)}$$

See Appendix 1 for the processing steps used to derive *Z*_SD_ using the QAA approach.

### Using contrast theory for estimating satellite-derived Secchi depth of turbid lakes and reservoirs from diffuse attenuation

As sunlight enters a waterbody, it is either absorbed or scattered by the constituents within the water column before emerging above the surface to be observed by an overlying sensor (i.e., either human eyes or a satellite/aircraft sensor). The absorption and scattering properties of the water column are specified in terms of the total absorption coefficient (*a*; m^−1^) and total scattering (*b*_b_; m^−1^), water backscatter (*b*_w_; m^−1^), and particle backscatter (*b*_bp_; m^−1^).

As a Secchi disk is lowered into the water at the surface and observed as it descends, the brightness and color from the disk will decrease as the depth increases until the contrast in brightness and color falls below the detection threshold of the observer’s eye (Aas et al., 2014). Lee et al. (2015) suggested that as a Secchi disk is lowered deeper and deeper into the water column its continued sighting by an observer means light is reflected from the disk to the human eye through an optically transparent window. Eventually, the difference between the disk and the surrounding water will diminish until the contrast in brightness falls below the detection threshold of the observer’s eye (Aas et al., 2014; Lee et al., 2015). Lee et al. (2015) used the term Contrast Theory to describe the relationship in which *K*_d_ is inversely proportional to Secchi disk depth.

### Statistical assessment and validation

The success of using the QAA and Contrast Theory to estimate *K*_D_ and predict *Z*_SD_ was evaluated by using a pair-wise comparison of algorithm residuals. The performance of the approach was compared using:

3 $$\textrm{Mean}\ \textrm{Absolute}\ \textrm{Error}\ \left(\textrm{MAE}\right)=\frac{\left[\sum_{n=1}^n\textrm{abs}\right(\left(\textrm{meas}\right)-\left(\textrm{pred}\right)\Big]}{n}$$

4 $$\textrm{bias}=\frac{\left[\sum_{\textrm{n}=1}^{\textrm{n}}\left(\textrm{meas}\right)-\left(\textrm{pred}\right)\right]}{n}$$

where *n* is the number of samples.

Other measures of performance included the standard error of the estimate (STDERR), coefficient of determination (*r*^2^), coefficient of variation (CV), and the regression slope (*m*) between predicted *Z*_SD_ values versus measured *Z*_SD_ values. These metrics were recommended by Seegers et al. (2018), who suggested that these statistics are necessary for aquatic remote-sensing validation efforts.

### Evaluating the recreational suitability of freshwater lakes and reservoirs using satellite-derived Secchi depths

The recreational suitability of the lakes and reservoirs in this study was evaluated using the *Z*_SD_ values derived from Landsat 8 spectral data and the water clarity framework (*Z*_SD-survey_) of Smith and Davies-Colley (1992) (Table 1).

**Table 1:**
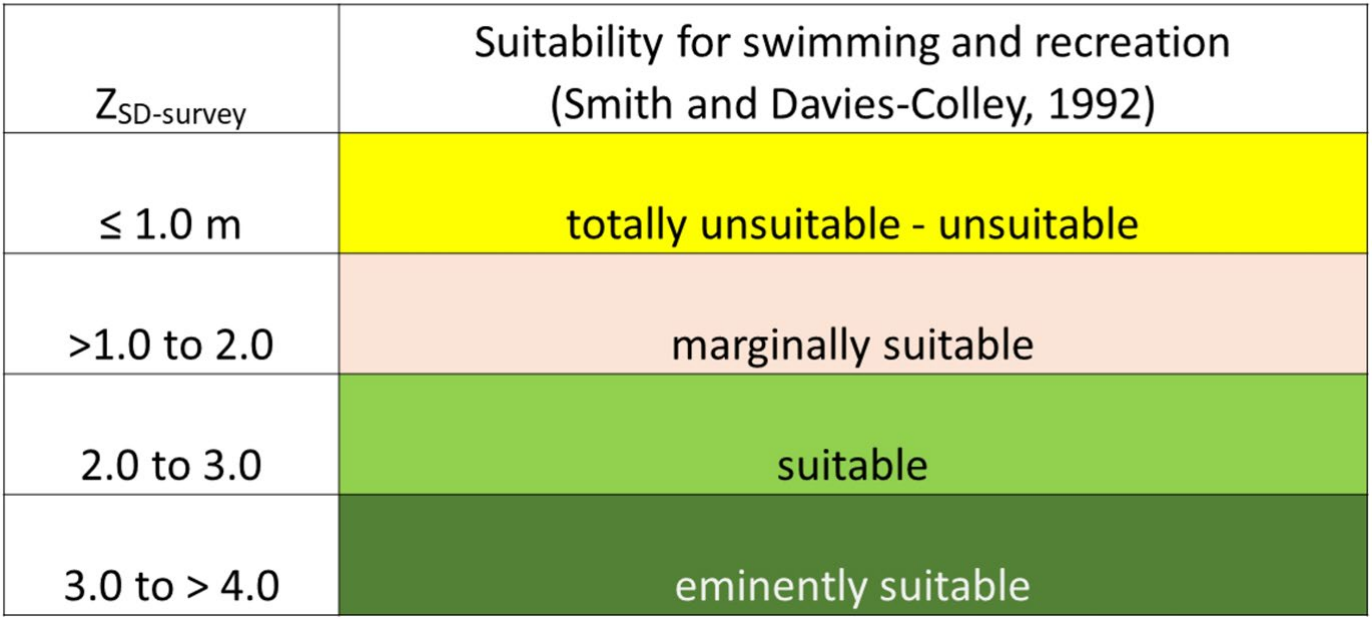
Suitability guidelines for recreation based on Secchi depth and public perception

The significance of the color scheme is to visually show the suitability for swimming and recreation. The most suitable and best waters are represented by the green hues. The color scheme grades to the pale hues which represent declining recreational suitability of lakes and reservoirs, with waterbodies in yellow completely not suitable

## Results and discussion

### Data acquisition

**Fig. 1:**
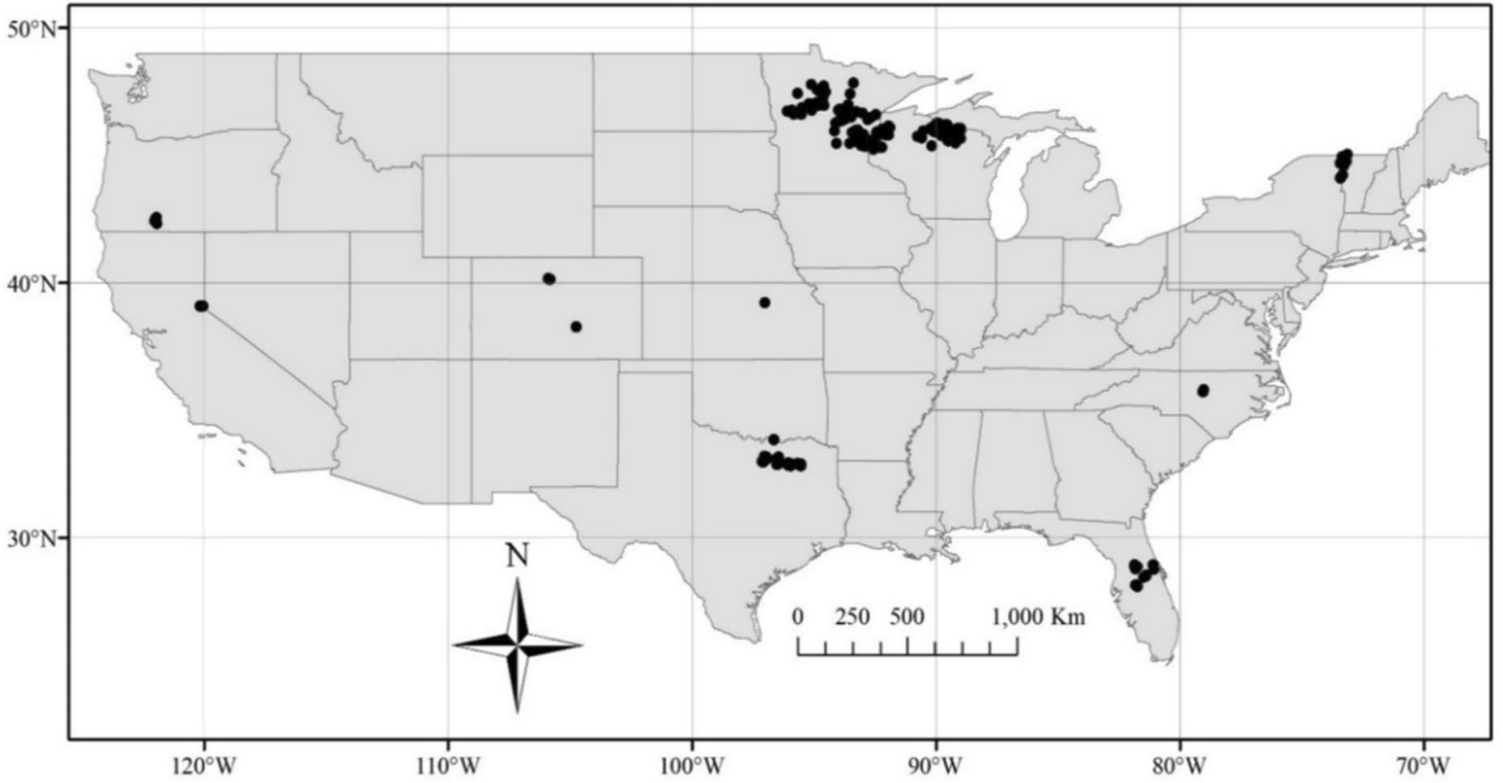
Geographic location of the 274 sites distributed across 10 states for this study

The query process identified 40 Landsat 8 images containing 274 field sites in 51 lakes and reservoirs from TX, KS, CO, OR, VT, MN, WI, CA, NC, and FL (Fig. 1; Table 4 in Appendix 2). Images covered June 2013–December 2019. Generally, satellite flyover dates occurred within 1 to 3 days of WQP, LAGOS-NE, and Aquasat field sampling dates (Soranno et al., 2017; Strelich, 2017). The water bodies ranged in size from eight (e.g., Morgan Lake, MN) to more than 50 million ha (e.g., Lake Tahoe, CA). *Z*_SD_ measurements were carried out with a 10–30-cm white disk at each field location. In situ Secchi measures ranged from 0.2 to 16.0 m with the shallowest measures in Lewisville Lake, TX and the deepest measures in Lake Tahoe, CA. Seasonally, 76% of measures were in summer (June–August), with 7, 1, and 15% of measures in autumn (September), winter (February), and spring (March/April), respectively. The seasonal in-situ sampling bias is typical of water-quality measures found in the Water Quality Portal and has been previously reported for temperature and chlorophyll-*a* (Papenfus et al., 2020; Schaeffer et al., 2018).

### Estimating the inherent optical properties of turbid lakes and reservoirs

In this study, we substituted wavelengths from Landsat 8 OLI sensor into QAA_v5 equations, noting the absence of a 667-nm band. This raises a primary concern of repurposing ocean color algorithms and terrestrial satellite sensors to become tools for monitoring optically complex inland water bodies. The concern is a difference in wavelengths between these remote-sensing systems may impair their ability to accurately retrieve IOPs and estimate water-quality indicators (e.g., water clarity). To address this concern, a synthesized hyperspectral dataset created by McKinna and Werdell (2019) and Werdell and McKinna (2019) was used to derive *R*_rs_ values. This dataset covered a range of optical environments from clear, oligotrophic oceanic to mesotrophic and eutrophic, and turbid coastal waters. Using *R*_rs_ values at 443, 481, and 655 nm, the wavelengths associated with Landsat 8 and 443-, 490-, and 670-nm wavelengths prescribed in QAA_v5 documentation *Z*_SD_ was predicted using the Contrast Theory based on *K*_d_ values estimated from QAA.

**Fig. 2:**
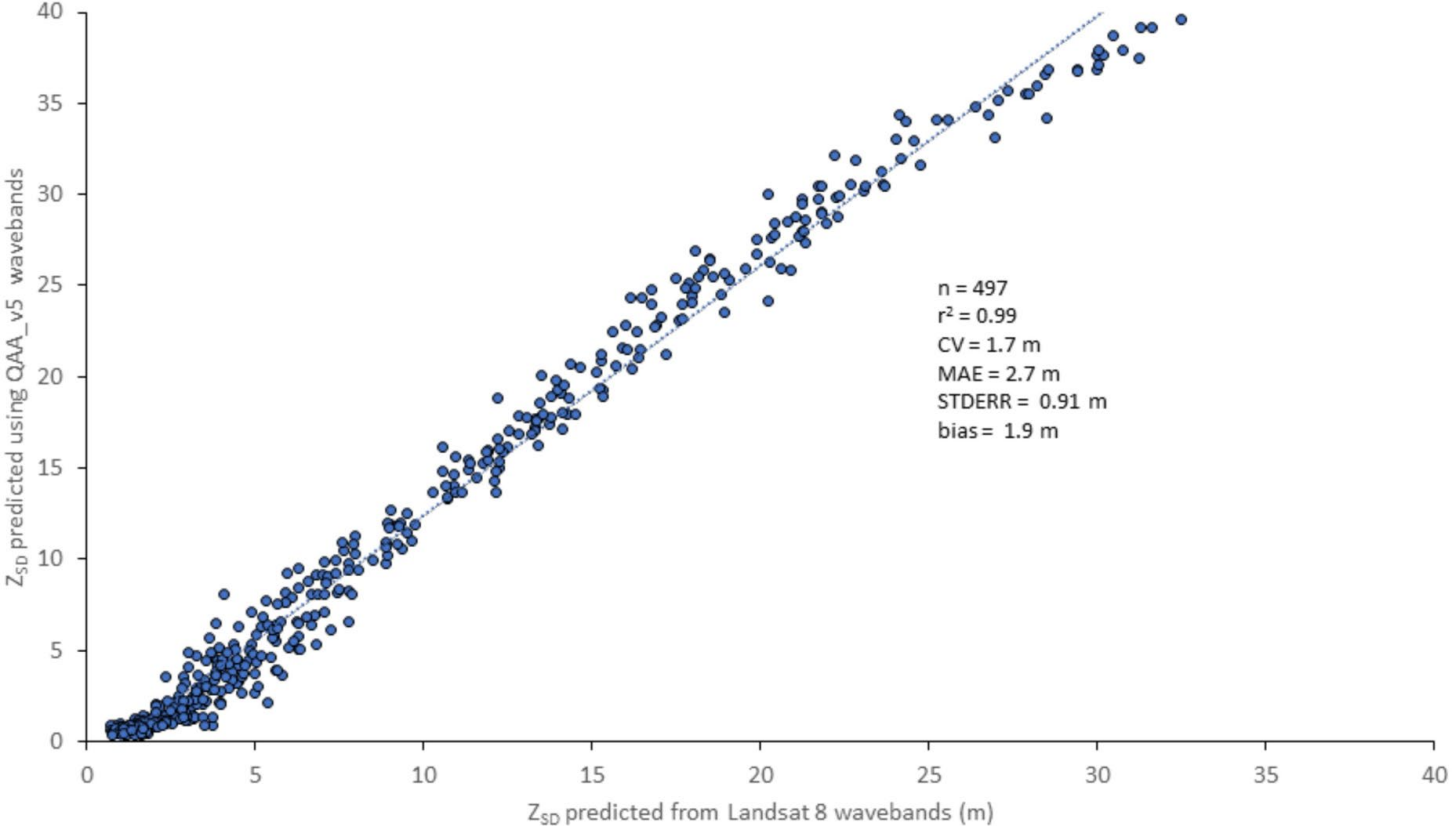
Relationship between *Z*_SD_ predicted using Landsat 8 wavebands and those prescribed in QAA_v5 using spectral data in McKinna and Werdell (2019)

A scatterplot of *Z*_SD_ values derived using QAA listed wavelengths and Landsat 8 wavelengths showed a linear relationship from less than 5 to approximately 40 m depth with a very strong *R*^2^ value (Fig. 2). The very strong agreement (*r*^2^ = 0.99) between *Z*_SD_ predicted from Landsat 8 bands and the QAA-prescribed wavebands indicate that OLI spectral data could be successfully substituted to predict *Z*_SD_ using QAA_v5 equations and Contrast Theory.

**Fig. 3:**
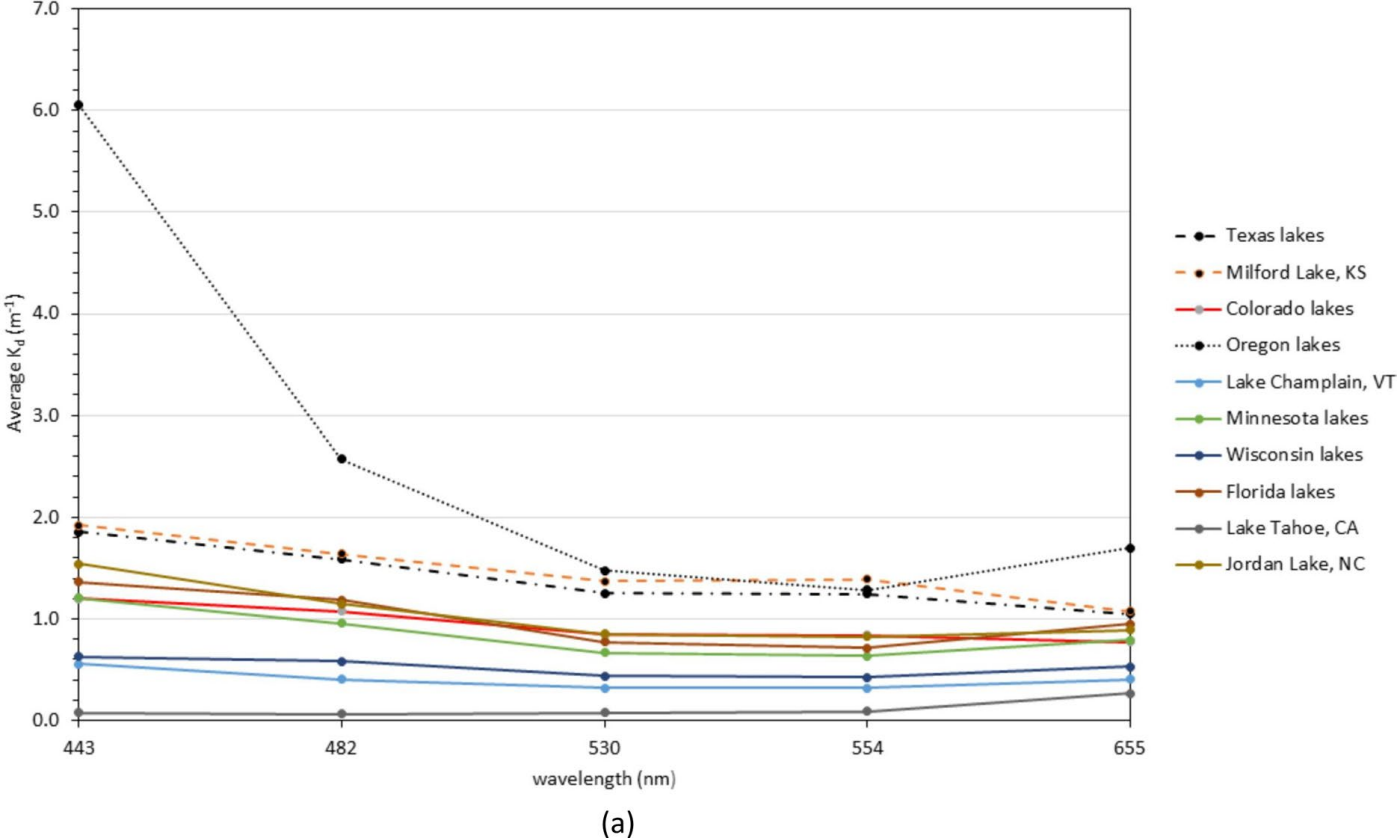

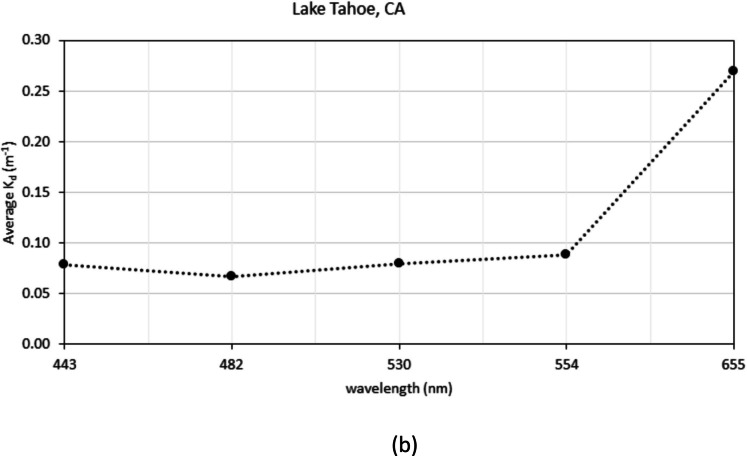
**a** Average *k*_d_ curve from the lakes in each region used in this analysis. **b** Average *k*_d_ curve for Lake Tahoe, CA showing the highest attenuation is trending to the red portion of the spectrum (i.e., 655 nm) and the lowest in the blue portion (i.e., 482 nm)

Based on the success of predicting *Z*_SD_ by substituting Landsat 8 spectral data into QAA_v5 and using Contrast Theory to predict water clarity, the IOP and *K*_d_ values were derived for the 274 sampling sites in the dataset. The average *K*_d_ values show the dataset ranged from 0.10 to approximately 6 m^−1^ (Fig. 3a, b). Geographically, OR lakes had the highest overall attenuation of all lakes. Spectrally, highest attenuations generally occurred in the Landsat 8 Coastal (band 1) and red (band 4) (centered at 443 and 655 nm, respectively). The lowest attenuations occur in the green band (band 3) (ranging from 530–554 nm) (Fig. 3a). However, Lake Tahoe, CA is the exception to this trend with the highest average *K*_d_ value occurring in the red (band 4) at 655 nm (Fig. 3b).

Using the assumption that the lower the attenuation value, the clearer the lake waters, Lake Tahoe, CA; the WI lakes; and Lake Champlain, VT constitute the clearest water bodies in the dataset. The lakes with the highest *k*_d_ values are the lakes in OR, KS (Milford Lake), and TX. The middle group consisted of MN lakes, CO lakes, FL lakes, and Jordan Lake, NC.

### Using contrast theory for estimating satellite-derived Secchi depth of turbid lakes and reservoirs from diffuse attenuation

**Fig. 4:**
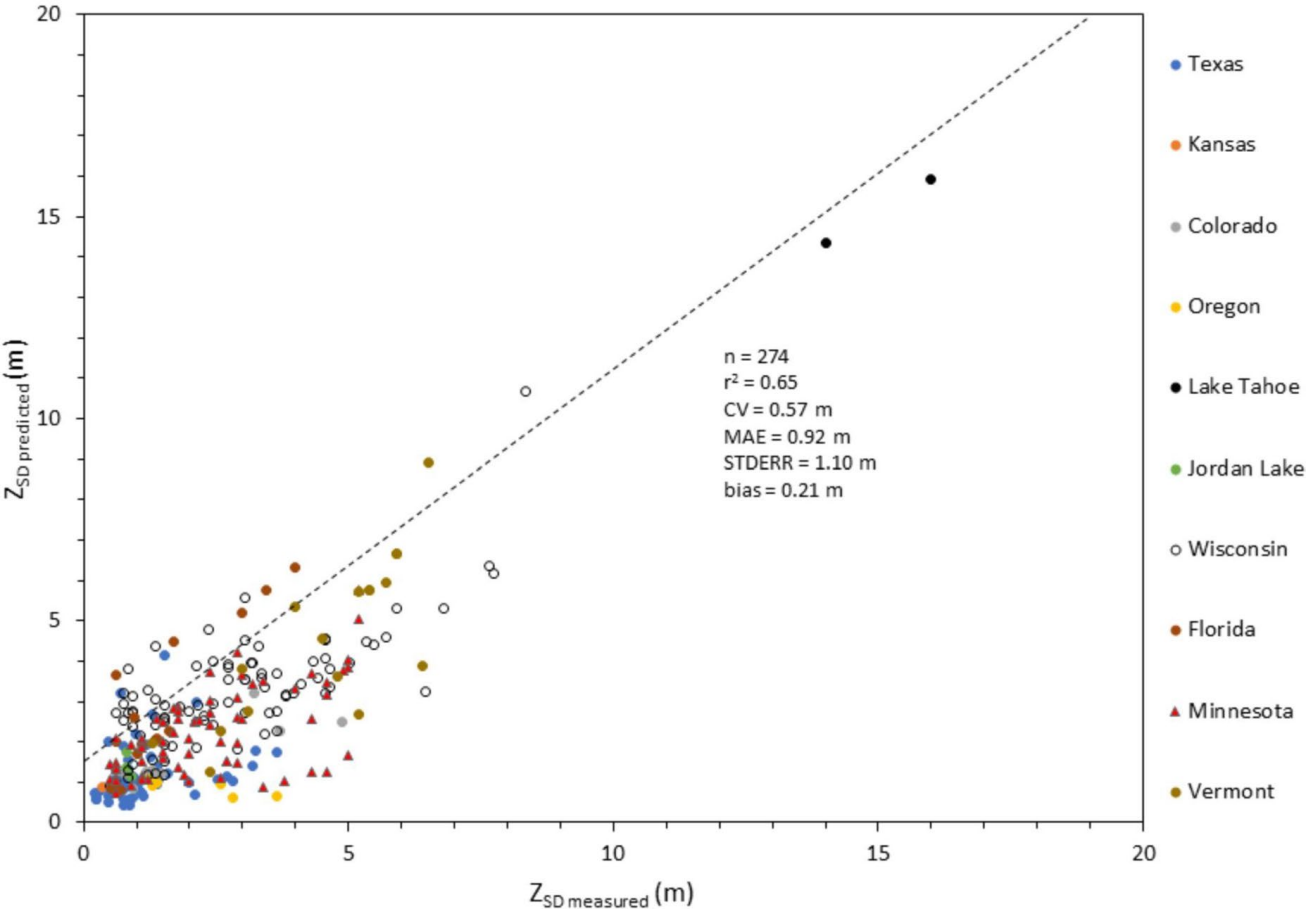
Scatterplot of *Z*_SD_ derived from L8 images processed using the QAA_v5/Contrast Theory approach (Lee et al., 2016) versus in situ Secchi depths (*n* = 274). The dotted line represents the 1:1 line

**Fig. 5:**
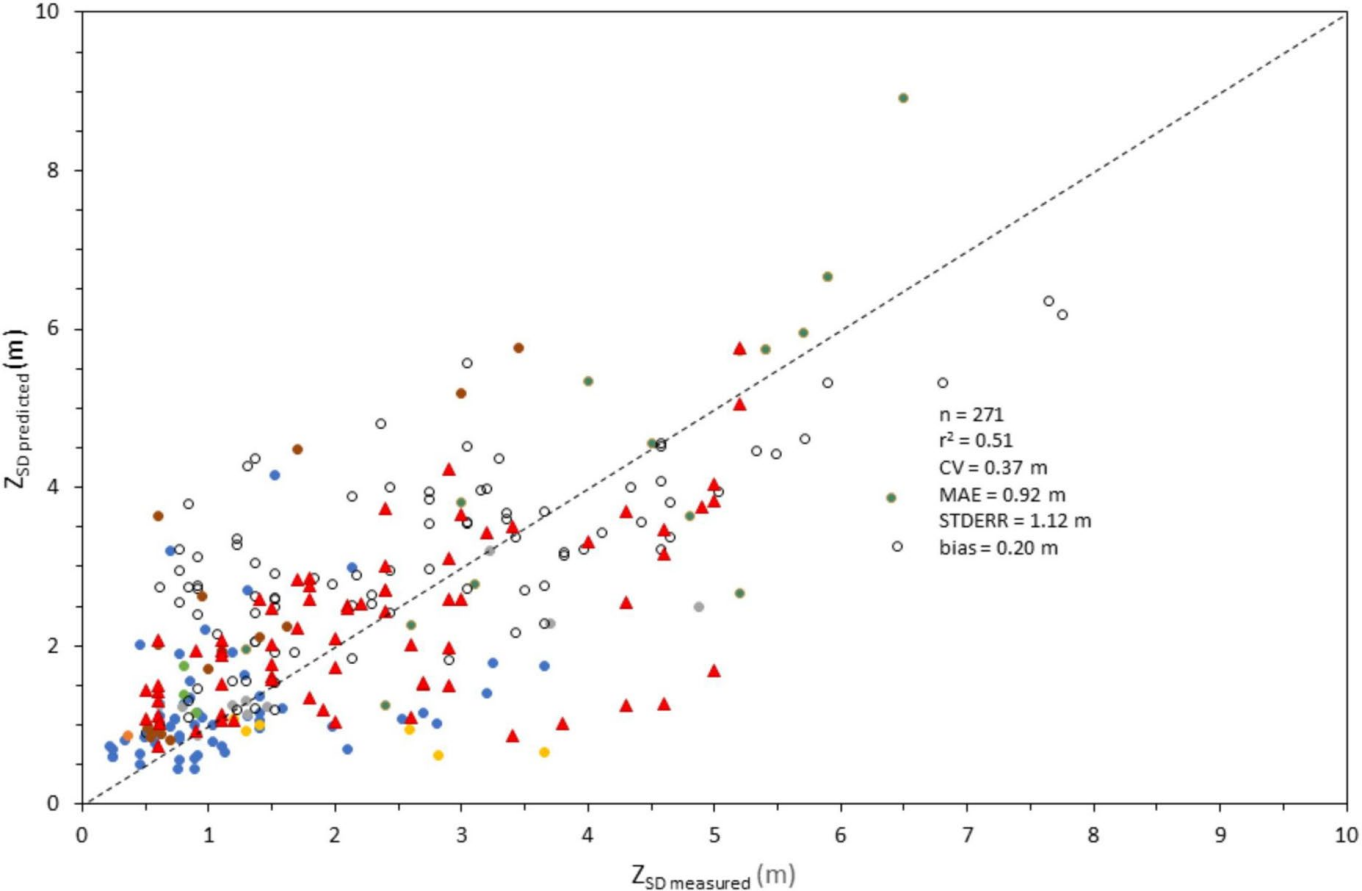
Scatterplot of *Z*_SD_ derived from L8 images processed using the QAA_v5/Contrast Theory approach (Lee et al., 2016), same legend as Fig. 4, for depths less than 10 m (*n* = 271). The dotted line represents the 1:1 line

A scatterplot of *Z*_SD_ predicted using QAA_v5/Contrast Theory mechanistic approach versus in situ Secchi depths from WQP, LEGOS, and Aquasat databases indicated a strong relationship between measured and predicted values. The graphical comparison also showed the entire dataset (*n* = 274) covered the Secchi depth range of less than 1 to 16 m with 46% of the measures less than 2 m (Fig. 4). Stephens et al. (2015) reported Secchi depth measures between less than 0.1 to 31.6 m across more than 14,000 US water bodies, with 50% of those measures being less than 2 m. Stephens et al. (2015) confirmed that the range validated in this study, using Landsat 8, covers the range of possible depths across US waters. The plot was also reconfigured to show Secchi values according to their geographical locations (Figs. 4 and 5).

Results indicated a bias value for both datasets of 0.21 and 0.20 m, respectively (Figs. 4 and 5). According to Seegers et al. (2018), bias offers a basic indication of the systematic direction (either over- or underestimating) prediction error. As a general rule, a bias value less than zero indicates a negative bias while bias values closer to one indicate less biased results (Seegers et al., 2018). The bias values suggest that the QAA/Contrast Theory approach has a minimal prediction bias. In addition, the MAE between *Z*_SD_ predicted from satellite and field observations was 0.92 m. This value is comparable to Page et al. (2019) which reported values from 0.25 to 0.67 m, based on a harmonized Sentinel-2 and Landsat 8 Secchi depth model for lakes across MN. The coefficient of variation (CV) is a normalized estimate of the data spread between Landsat 8 and in situ *Z*_SD_ values around the mean (Seegers et al., 2018). In this study, CV values were 0.37 m for the entire dataset and 0.52 m for those stations with Secchi values less than 8 m. The STDERR was 1.1 m for both datasets and *r*^2^ as 0.65 for the entire dataset and 0.51 for those stations with Secchi values less than 8 m. In summary, the performance metrics suggested that the QAA/Contrast Theory approach reasonably predicts slightly biased accurate *Z*_SD_ values from satellite spectral data.

### Evaluating the recreational suitability of freshwater lakes and reservoirs using satellite-derived secchi depths

**Fig 6:**
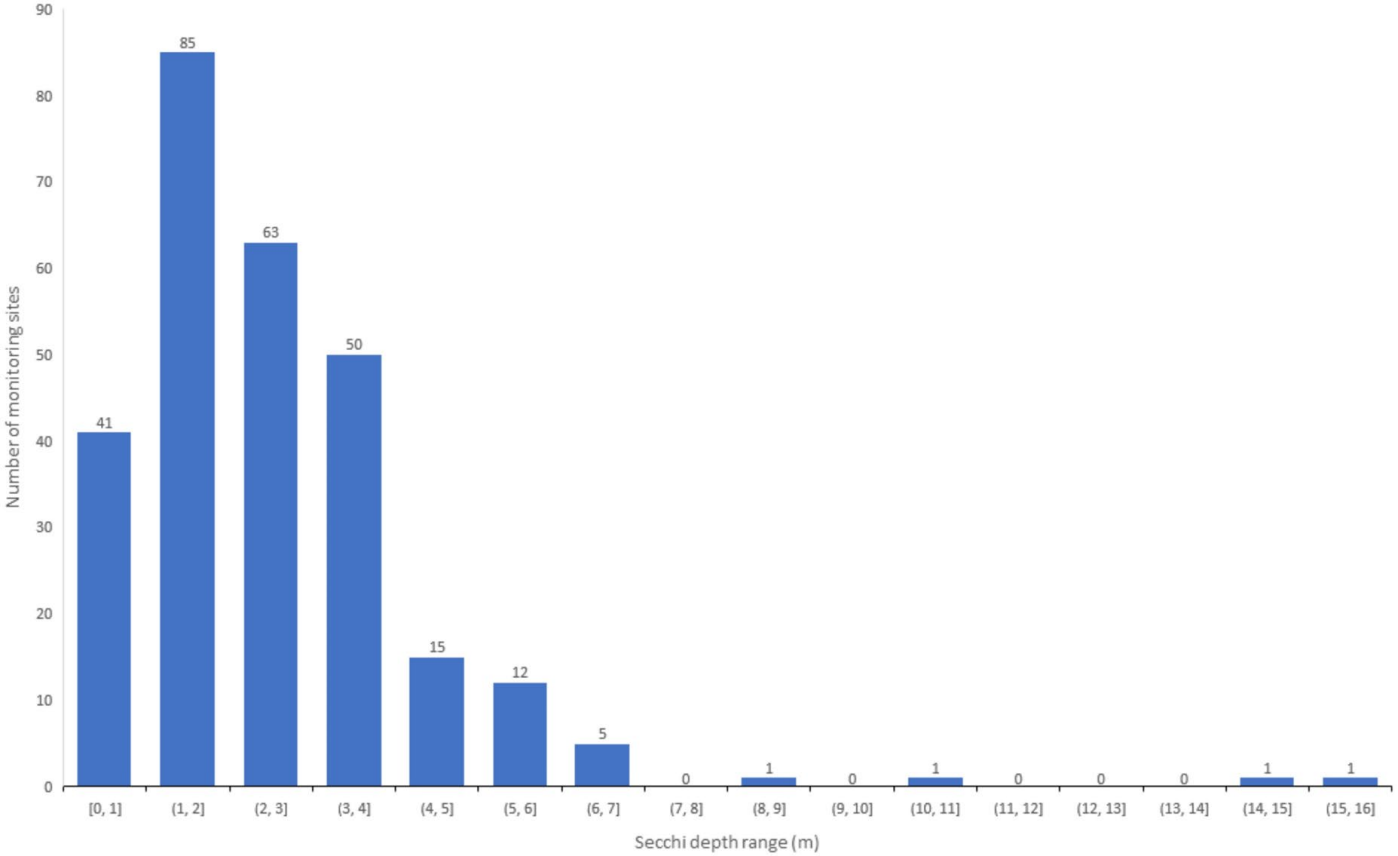
Histogram of the number of monitoring sites with Secchi disk depth ranges from less than 1 to 16 m

The framework for evaluating the suitability of water bodies in our dataset to provide recreational benefits is based on the human perception that clear, non-turbid waters are visually appealing (Angradi et al., 2018; Smith et al., 1995; Smith & Davies-Colley, 1992; Table 2). Secchi depths predicted by the QAA/Contrast Theory approach were binned according to the suitability framework presented by Smith and Davies-Colley (1992) (Fig. 6 and Table 2).

**Table 2:** Geographic distribution of the suitability for swimming and recreation based on the predicted Secchi depth and  the Smith and Davies-Colley (1992) framework. Included also are the number of sites, in parentheses, within each state that met the suitability criteria

**Totally unsuitable–unsuitable** (*n* = 41)	**Marginally suitable** (*n* = 85)	**Suitable** (*n* = 63)	**Eminently suitable** (*n* = 86)
Secchi depth range (0–1 m)	Secchi depth range (1–2 m)	Secchi depth range (2–3 m)	Secchi depth range (3 – >4 m)
Texas (22)	Minnesota (29)	Wisconsin (30)	Wisconsin (48)
Florida (6)	Texas (27)	Minnesota (21)	Minnesota (14)
Oregon (5)	Wisconsin (13)	Florida (4)	Lake Champlain (12)
Wisconsin (2)	Colorado (6)	Lake Champlain (3)	Florida (5)
Minnesota (5)	Florida (3)	Texas (3)	Lake Tahoe (2)
Colorado (1)	Jordan Lake (3)	Colorado (2)	Colorado (1)
	Lake Champlain (2)		Texas (4)
	Oregon (1)		
	Kansas (1)

Secchi depths at individual locations were binned to create a range of values at 1-m intervals (Fig. 6) and categorized according to public perception based on the suitability criteria (Tables 1 and 2). Results show that lakes and reservoirs in CO, FL, TX, MN, VT, and WI cover all categories of recreational suitability based on water clarity. All sites, (numbers are parentheses) in OR (6), Jordan Lake (NC; 3), and Milford Lake (KS; 1) were categorized as totally unsuitable to unsuitable or marginally suitable for recreation. In TX (49 of 56 sites; 87.5%), WI (15 of 93 sites; 16.1%), MN (34 of 68 sites; 50.0%), CO (7 of 10 sites; 70.0%), and FL (9 of 18 sites; 50.0%) several sites were categorized as totally unsuitable to unsuitable or marginally suitable for recreation. Lake Champlain (VT) was the exception with only two (of 17 sites; 11.8%) categorized as marginally suitable for recreation. In comparison, the majority of lakes and reservoirs in WI (78 of 93 sites; 83.9%), MN (34 of 68 sites; 50.0%), and Lake Champlain (VT) (15 of 17 sites; 88.2%) were categorized as suitable to eminently suitable. The FL (9 of 18 sites; 50.0%), CO (3 of 10 sites; 30.0%), and TX (7 of 56 sites; 12.5%) sites were categorized as suitable to eminently suitable. For Lake Tahoe (CA) (2 of 2 sites; 100%) were categorized as eminently suitable.

As an alternative approach, the individual Secchi values within each state were aggregated and averaged to produce a generalized state average. These state averages were also categorized for their suitability for recreation and compared with the results from the more detailed Secchi data (compare Tables 2 and 3). Results showed that the alternative approach indicated that the majority of the lakes and ponds in the averaged dataset would be very acceptable for swimming as 66% (*n* = 181) of all sites fell into the eminently suitable–suitable categories for recreation. These sites were located in the Great Lakes region (WI and MN), Lake Champlain, and Lake Tahoe. The remaining 34% (*n* = 93) of water bodies, however, were spread between the totally unsuitable to marginally suitable categories (Table 3). This suggested that perception of recreational suitability for swimming was problematic but that perhaps these water bodies were more appropriate for providing other services such as fishing.

**Table 3:**
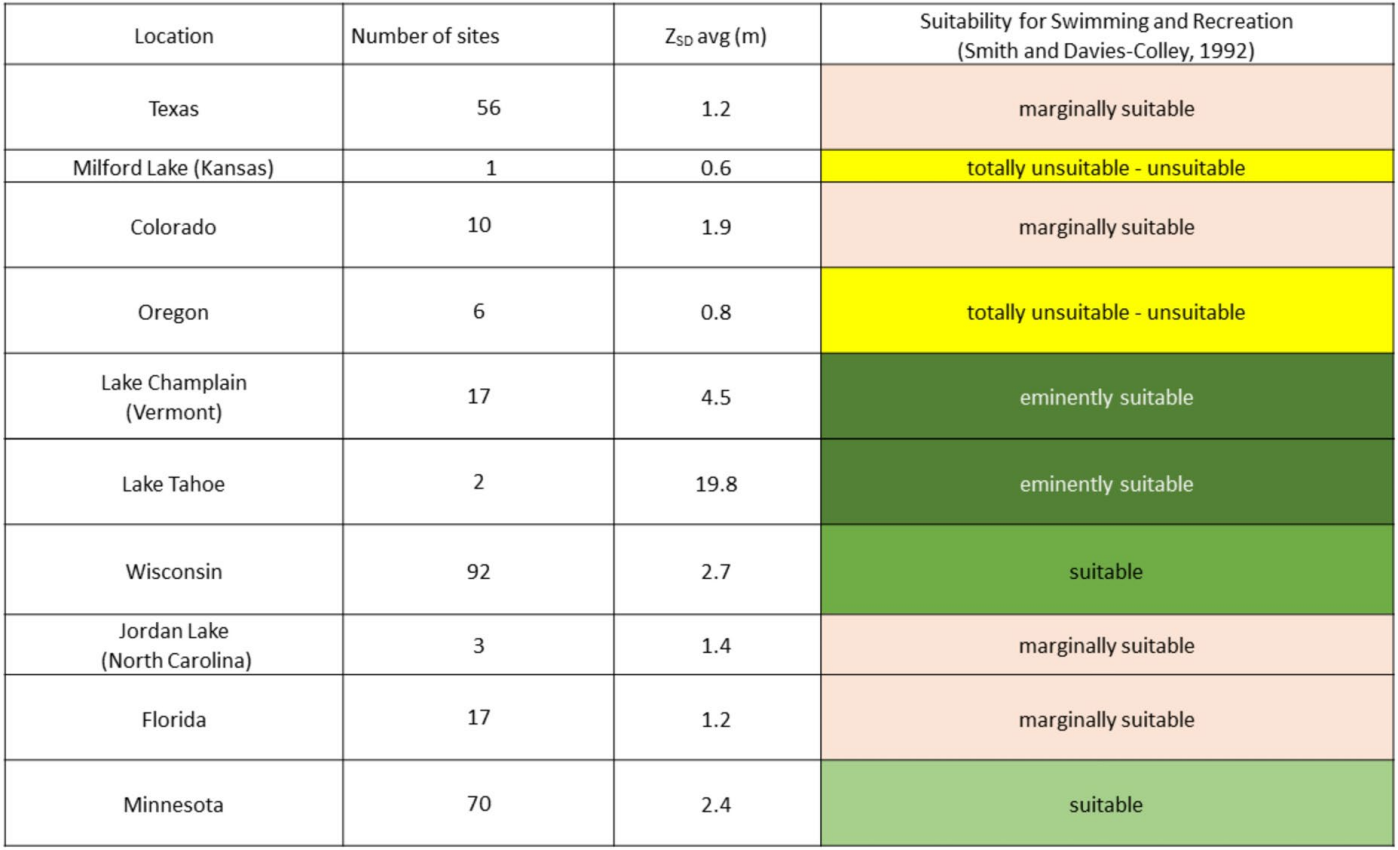
Regional summary of recreational suitability based on Landsat 8 *Z*_SD_ and public perception

The significance of the color scheme is to visually show the suitability for swimming and recreation. The most suitable and best waters are represented by the green hues. The color scheme grades to the pale hues which represent declining recreational suitability of lakes and reservoirs, with waterbodies in yellow completely not suitable

## Conclusions

Managers need a way to predict beforehand whether their proposed water-management strategies will produce significant results or be worth the cost. Water clarity (i.e., Secchi data) derived from remote-sensing could certainly be used as an effective tool to provide information for informed data analysis at national, state, tribal, and watershed scales. This type of information could also be used as an important link between doing water-quality restoration activities and determining their effectiveness.

We found that the clarity of inland lakes and reservoirs can be accurately derived using no-cost, high-resolution satellite data. We used this information to assess the suitability of freshwater lakes and reservoirs in our dataset for public recreation. The implications are that satellites engineered for terrestrial applications can be successfully used with traditional ocean-color algorithms and methods to study the water quality of freshwater environments. Furthermore, operational land-based satellite sensors such as the OLI have the temporal repeat cycles, spectral resolution, wavebands, and signal-to-noise ratios to be repurposed to monitor water quality for public use and consumption and trophic status of complex inland waters.

## Data Availability

The remote-sensing and water quality datasets associated with this study can be obtained from the US Environmental Protection Agency Environmental Dataset Gateway (EDG) at https://edg.epa.gov/metadata/catalog/main/home.page.

## Appendix 1 Processing steps used to derive *Z*_SD_ using the QAA approach and Landsat 8 spectral data

The steps below are taken from Lee et al. (2016).

Step 1. Above-water remote-sensing reflectance (*R*(0+)) was converted to subsurface remote-sensing reflectance (*R*(0−)). Units are sr^−1^.
  5 $$R\left(0-\right)=\frac{R\left(0+\right)\left(\lambda \right)}{0.52+1.7R\left(0+\right)\left(\lambda \right)},\textrm{where}\ \left(\lambda \right)\ \textrm{is}\ \textrm{wavelength}.$$
Step 2. Gordon et al. (1988) indicated that *R*(0−) is a function of the ratio of *b*_b_/*a* + *b*_b_ which can be expressed as:
  6 $$R(0-)=(g_{0}+g_{1}\;b_{b}/\;(a+b))\;b_{b}/\;(a+b_{b})$$
  where *g*_0_ = 0.089 sr^−1^ and *g*_1_ = 0.125 sr^−1^ (Lee et al., 2002)
  This quadratic function yields:
  7 $$\begin{aligned}&u\left(\lambda \right)=\frac{-{g}_0+\sqrt{{\left({g}_0\right)}^2}+4{g}_1\times R\left(0-\right)\left(\lambda \right)}{2{g}_1}\\&\qquad\quad\;\; \mathrm{where}\quad u\left(\lambda \right)={b_b}/{\left(a+{b}_b\right)}\end{aligned}$$
Step 3. The QAA process starts with an estimation of total absorption (*a*_t_) at a reference wavelength (*λ*_0_) which is expressed as:
  8 $$a\left({\lambda}_0\right)={a}_w\left({\lambda}_0\right)+\Delta a\left({\lambda}_0\right)$$
  where *a*_w_ is the absorption coefficient of pure water and assumed to be a constant (Lee et al., 2016) and ∆*a*(*λ*_0_), which is empirically estimated from the *R* (0−) spectrum, represents the contributions from dissolved and suspended sediments (Lee et al., 2002). Lee et al. (2016) suggested that for an easy processing of L8 images, 554 nm should be used as the representative wavelength for L8 band 3:
  9 $${a}_{t(554)}={a}_{w(554)}+0.39\times \frac{R\left(0-\right)554}{\left(R\left(0-\right)443+R\left(0-\right)481\right)}$$
  where *a*_w (554)_ is the absorption coefficient of pure water at 554 nm which is 0.0596 m^−1^ (Pope & Fry, 1997).
Step 4. Once *a*_*t*_(*λ*_0_) is known, the total backscatter (*b*_b_(*λ*_0_)) can be derived from the particle backscatter at the reference wavelength (*b*_bp_(554)):
  10 $${b}_{\textrm{bp}}(554)=u(554)\times{a_t(554)}/{\left(1-u(554)\right)}-{b}_{\textrm{bw}}(554)$$
  where *b*_bw_(554) is the backscattering coefficient of pure water at 554 nm, which is 0.0014 m^−1^ (Smith & Baker, 1981).
Step 5. The *b*_bp_ values at other wavelengths (*λ*) are estimated following a power-law function (Gordon & Morel, 1983)
  11 $${b}_{\textrm{bp}}\left(\lambda \right)={b}_{\textrm{bp}}(554)\times {\left({554}/{\lambda}\right)}^{\eta }$$
  The exponent η is empirically estimated from the *R*(0−) spectrum (Lee et al., 2005) by
  12 $$\eta =2.2\Big(1.12\times \exp \left(-0.9\times {R{\left(0-\right)}_{443}}/{R{\left(0-\right)}_{554}}\right)$$
Step 6. Once *R*(0−)(*λ*), *u*(*λ*), *a*(*λ*), and *b*_bp_(*λ*) are known, the total absorption *a*_t_(*λ*) can be derived using
  13 $$a\left(\lambda \right)=\left(1-u\left(\lambda \right)\right)\times {\left({b}_{\textrm{bw}}\left(\lambda \right)+{b}_{\textrm{bp}}\left(\lambda \right)\right)}/{u\left(\lambda \right)}$$
Step 7. Total backscatter *b*_b_(*λ*) can be derived using
  14 $${b}_b\left(\lambda \right)={b}_b\left(\lambda \right)+{b}_{bp(554)}\times {\left(554/\lambda \right)}^{\eta }$$
Step 8. The diffuse attenuation coefficient (*k*_d_) is the coefficient at the transparent window of a waterbody within the visible domain (410–665 nm) which can be modeled as (Lee et al., 2013):
  15 $$\begin{aligned}{K}_d\left(\lambda \right)=&\ \left(1+{m}_0\times {\theta}_s\right)\times {a}_t\left(\lambda \right)\\&+\left(1-\gamma \times \left({b_{bw}\left(\lambda \right)}/{b_b\left(\lambda \right)}\right)\ \right)\\&\times {m}_1\times (1-{m}_2\times \mathrm{EXP}\\&\left(-10.8\times {a}_t(\lambda)\right)\times {b}_b(\lambda )\end{aligned}$$
  where *m*_0_ = 0.005, *θ*_s_ = solar zenith angle (in degrees), *γ* = 0.265, *m*_1_ = 4.26, and *m*_2_ = 0.52 (Lee et al., 2016).
Step 9. Secchi depth (*Z*_SD_) is inversely proportional to *K*_d_ and can be expressed as
  16 $${Z}_{\textrm{SD}}=\frac{1}{2.5\operatorname{Min}\left({k}_d\right)}\ln \left[\left(\frac{t^2}{n^2}\right)\frac{\left|{r}_{\textrm{T}}-{r}_{\textrm{w}}^{pc}\right|}{c_{\textrm{t}}\left(0-\right)}\right],$$
  where Min*(k*_d_*)* = minimum diffuse attenuation at the transparent window within the visible domain (400–700 nm; Lee et al., 2016)

- $$\left(\frac{t^2}{n^2}\right)=\frac{\textrm{radiance}\ \textrm{transmittance}\ \textrm{across}\ \textrm{the}\ \textrm{water}-\textrm{air}\ \textrm{interface}}{\textrm{refractive}\ \textrm{index}\ \textrm{of}\ \textrm{water}}=0.52$$ (Mobley, 1994)
- *r*_T_ = the downwelling irradiance just below the water surface = 0.27 (Preisendorfer, 1986)
- *r*_w_^pc^ = the subsurface reflectance ≡ *r*_rs_
- *c*_*t*_ (0−) = the contrast threshold for sighting a Secchi disk below the water surface; Weber contrast (Johnsen et al., 2011)

17 $${c}_t\left(0-\right)=\frac{\operatorname{Min}\left({k}_{\textrm{d}}\right)\left(a+{b}_{\textrm{b}}\right)}{\left(a+{b}_{\textrm{b}}\right)}$$

## Appendix 2

**Table 4:** Landsat 8 scene identifiers and sample sites used in this study

Scene ID	State	Number of sites
LC80260372015194LGN01 LC80270362013163LGN01 LC80270362014070LGN01 LC80270372013163LGN01 LC80270372014118LGN01 LC80270372015217LGN01 LC80270372016124LGN01 LC80270372016188LGN01 LC80270372016204LGN01 LC80270372016124LGN01	TX	56
LC80280332016179LGN01	KS	1
LC80330332014160LGN01 LC80330332015259LGN02 LC80330332016166LGN01 LC80330332016198LGN01 LC80340322016189LGN01	CO	10
LC80450302015199LGN01	OR	6
LC80140292014155LGN00 LC80140292014187LGN00 LC80140292014235LGN00 LC80140292015254LGN00 LC80140292014267LGN00 LC80140292019080501T1 LC80140292019100801T1 LC80114029201912111T1	VT	20
LC80290272016234LGN01 LC80280272016243LGN01 LC80270282018225LGN00	MN	70
LC80240282016247LGN01 LC80240282015228LGN01 LC80250282016174LGN01 LC80270282018225LGN00	WI	92
LC80430332016108LGN01 LC80430332016204LGN01 LC80430332016044LGN01 LC80430332016076LGN01 LC80430332016236LGN01 LC80430332016220LGN01	CA	6
LC8016040201401LGN01	FL	17
LC80160352015092LGN00 LC80160352014345LGN00	NC	3
